# An *Ex Vivo* Model for Anti-Angiogenic Drug Testing on Intact Microvascular Networks

**DOI:** 10.1371/journal.pone.0119227

**Published:** 2015-03-05

**Authors:** Mohammad S. Azimi, Leann Myers, Michelle Lacey, Scott A. Stewart, Qirong Shi, Prasad V. Katakam, Debasis Mondal, Walter L. Murfee

**Affiliations:** 1 Department of Biomedical Engineering, Tulane University, New Orleans, Louisiana, United States of America; 2 Department of Biostatistics & Bioinformatics, Tulane University, New Orleans, Louisiana, United States of America; 3 Department of Mathematics, Tulane University, New Orleans, Louisiana, United States of America; 4 Department of Pharmacology, Tulane University, New Orleans, Louisiana, United States of America; University of Bari Medical School, ITALY

## Abstract

New models of angiogenesis that mimic the complexity of real microvascular networks are needed. Recently, our laboratory demonstrated that cultured rat mesentery tissues contain viable microvascular networks and could be used to probe pericyte-endothelial cell interactions. The objective of this study was to demonstrate the efficacy of the rat mesentery culture model for anti-angiogenic drug testing by time-lapse quantification of network growth. Mesenteric windows were harvested from adult rats, secured in place with an insert, and cultured for 3 days according to 3 experimental groups: 1) 10% serum (angiogenesis control), 2) 10% serum + sunitinib (SU11248), and 3) 10% serum + bevacizumab. Labeling with FITC conjugated BSI-lectin on Day 0 and 3 identified endothelial cells along blood and lymphatic microvascular networks. Comparison between day 0 (before) and 3 (after) in networks stimulated by 10% serum demonstrated a dramatic increase in vascular density and capillary sprouting. Growing networks contained proliferating endothelial cells and NG2+ vascular pericytes. Media supplementation with sunitinib (SU11248) or bevacizumab both inhibited the network angiogenic responses. The comparison of the same networks before and after treatment enabled the identification of tissue specific responses. Our results establish, for the first time, the ability to evaluate an anti-angiogenic drug based on time-lapse imaging on an intact microvascular network in an *ex vivo* scenario.

## Introduction

Models that mimic angiogenesis, defined as the growth of new blood vessels from existing vessels, are extremely valuable for the investigation of underlying mechanisms and the pre-clinical development of therapies. Historically, *in vitro* models have proven crucial for mechanistic investigation of intra-cellular signaling and cell-cell interactions [1]. Two-dimensional culture or co-culture systems, however, are limited in their complexity and the physiological relevance can be unclear. Recognition of the need to incorporate the multi-scale complexity of a real microvascular scenario, i.e., cells, vessels and network, has motivated the development of three-dimensional culture systems [1], *ex vivo* tissue explant models [2], microfluidic platforms [3,4], and the use of integrated computational approaches [5]. Since angiogenesis involves multiple cell types and is related to the growth of other systems, such as lymphatic networks [6,7], a need still exists for a model of angiogenesis from intact microvascular networks that more closely reflects an *in vivo* scenario.

Our laboratory recently introduced the rat mesentery culture model and demonstrated that blood and lymphatic microvascular networks remain intact *ex vivo*. We also showed that this model could be used to investigate pericyte-endothelial cell interactions during capillary sprouting [8]. The objective of this study was to demonstrate the efficacy of the rat mesentery culture model for anti-angiogenic drug testing by time-lapse quantification of network growth. An advantage of using rat mesentery tissue is that it is self-contained and does not require embedding into a matrix. The thickness of the mesentery tissue (20–40 μm) [9] also enables observation of entire intact networks. By simply securing the tissue on the bottom of a culture well, labeling live endothelial cells with BSI-lectin and using epifluorescent imaging, we demonstrate that vessel density and capillary sprouting can be quantified in a network before and after angiogenesis stimulation. Application of this method for anti-angiogenic drug testing is supported by inhibition with sunitinib, a tyrosine kinase inhibitor targeting VEGFR-2, and bevacizumab, a known VEGF-A inhibitor. Our results suggest that the rat mesentery culture model offers a pre-clinical drug-screening platform that enables observation of drug effects within an intact microvascular network with innate hierarchical pattern.

## Materials and Methods

### Rat Mesentery Culture Model

All animal experiments were approved by Tulane University’s Institutional Animal Care and Use Committee. Rat mesenteric tissues were harvested and cultured according to our previous description [8]. Adult male Wistar rats (325–349 g) were anesthetized via intramuscular injection with ketamine (80 mg/kg body weight) and xylazine (8 mg/kg body weight). The mesentery was aseptically exteriorized [10] and rats were euthanized by intracardiac injection of 0.2 ml Beuthanasia. Then, mesenteric windows, defined as the thin, translucent connective tissues found between artery/vein pairs feeding the small intestine, were harvested, starting from the ileum. Tissues were immediately rinsed in sterile phosphate-buffered saline (PBS; Gibco; Grand Island, NY) with CaCl_2_ and MgCl_2_ at 37°C, and immersed in sterile minimum essential media (MEM; Gibco; Grand Island, NY) containing 1% Penicillin-Streptomycin (PenStrep; Gibco; Grand Island, NY). They were then transferred to individual wells in either a 6-well or 12-well culture plate. For drug studies, each tissue was quickly spread out on the bottom of a well and secured in place with a commercially available insert (CellCrown; Sigma-Aldrich; St. Louis, MO) with polycarbonate filter and covered with 3 ml of MEM containing 1% PenStrep. Tissues were cultured in standard incubator conditions (5% CO_2_).

### Time-Lapse Imaging and Quantification of Angiogenesis

Tissues (n = 16 tissues; 4 tissues harvested from 4 rats each) were cultured in MEM + 10% fetal bovine serum (FBS; Gibco; Grand Island, NY). On day 0, the media in each well was supplemented with BSI-Lectin conjugated to FITC (1:40; Sigma-Aldrich; St. Louis, MO). Tissues were then incubated for 30 minutes under standard culture conditions. After the incubation, the lectin-supplemented media was removed, and tissues were washed with lectin-free media. Labeling with BSI-Lectin identified all blood and lymphatic vessels and blood vessels were identified from lymphatics based on their morphology and network structure [11]. “Before” images of the microvascular networks were obtained per tissue. Tissues were then returned and cultured according to their experimental group for 3 days. On day 3, tissues were again labeled with BSI-lectin and imaged. Positioning of the motorized microscope stage insured that the same microvascular network region was re-imaged. The number of vessels per vascular area and the number of capillary sprouts per vascular area were quantified per tissue from 4x images of randomly selected network regions per tissue. Two to four fields of view were arbitrarily selected from the vascularized area to be representative of the microvascular networks. Blood vessel segments were defined as lectin-positive blood endothelial cell segments present between two branch points and capillary sprouts were defined as blind-ended segments originating from a host vessel.

### Immunohistochemistry

Additional tissues were harvested, cultured and labeled according to the following steps. **Lectin + NG2**: 1) 1:100 rabbit polyclonal NG2 antibody (Millipore; Billerica, MA); 2) 1:100 goat anti-rabbit CY3-conjugated antibody (GAR-CY3) and 5% NGS. **Lectin + BrdU**: 1) 2 h incubation in 1mg/ml BrdU (Sigma-Aldrich; St. Louis, MO) supplemented media; 2) 6 M HCl for 1 h at 37°C to denature the DNA; 3) 1:100 mouse monoclonal anti-BrdU (Dako; Carpinteria, CA); 4) 1:100 GAM-CY3 Fab fragments (Jackson ImmunoResearch Laboratories; West Grove, PA). **Live/Dead**: 1) 1:400 Calcein AM and 1:200 EthD-1 (Molecular Probes; Grand Island, NY) for 10 min at 37°C. Lectin labeling was performed as described above. Tissues were fixed with methanol fixation at -20°C for 30 min. All antibodies were diluted in antibody buffer solution (PBS + 0.1% saponin + 2% bovine serum albumin).

### Drug Testing Studies

To validate the ability of the time-lapse imaging method to evaluate the anti-angiogenic effects for a given drug treatment, two drug studies were conducted. In the first study, mesenteric windows were harvested from adult male Wistar rats and cultured for 3 days according to the two experimental groups: 1) 10% serum (n = 8 tissues from 4 rats), and 2) 10% serum + sunitinib (Cayman Chemical Company; San Diego, CA) (5 μM; n = 8 tissues from 4 rats). Sunitinib was selected because it is a multi-targeted receptor tyrosine kinase inhibitor and has commonly been used to inhibit endothelial cell dynamics associated with angiogenesis in both *in vitro* and *in vivo* assays [12–14]. The concentration for sunitinib was selected based on previously published *in vitro* studies [15,16].

For the second drug exposure study, mesenteric windows were harvested from adult male Wistar rats and cultured for 3 days according to the two experimental groups: 1) 10% serum (n = 8 tissues from 4 rats), and 2) 10% serum + bevacizumab (Genentech, San Francisco, CA) (10 μg/ml; n = 8 tissues from 4 rats). The bevacizumab, commercially known as Avastin, was selected for this study for its well-known anti-VEGF properties [17]. The number of vessel segments per vascular area and the number of capillary sprouts per vascular area were quantified per tissue from 4x images of randomly selected network regions per tissue. To eliminate any false negative drug responses in drug treated tissues, tissues were analyzed only when corresponding tissues from the same rat showed growth caused by serum treatment.

### Image Acquisition

Images were acquired using 4x (dry, NA = 0.1), 10x (dry, NA = 0.3), 20x (oil, NA = 0.8), and 60x (oil, NA = 1.4) objectives on an inverted microscope (Olympus IX70) coupled with a Photometrics CoolSNAP EZ camera.

### Statistical Analysis

Data were analyzed in terms of change in number of vessel segments and number of capillary sprouts between day 0 and day 3. Mixed model regression methods were used to determine whether the change from day 0 to day 3 was significant and whether the amount of change was significantly different between drug dosages. Mixed model methods were used to control the non-independence of change scores measured on the same subject; reported means are adjusted for this correlation. Where possible, alternative nonparametric analyses were used to confirm the findings. All analyses were conducted using SAS version 9.3.

## Results

### Time-Lapse Imaging Enables Tissue Specific Quantification of Microvascular Network Growth

**Fig 1:**
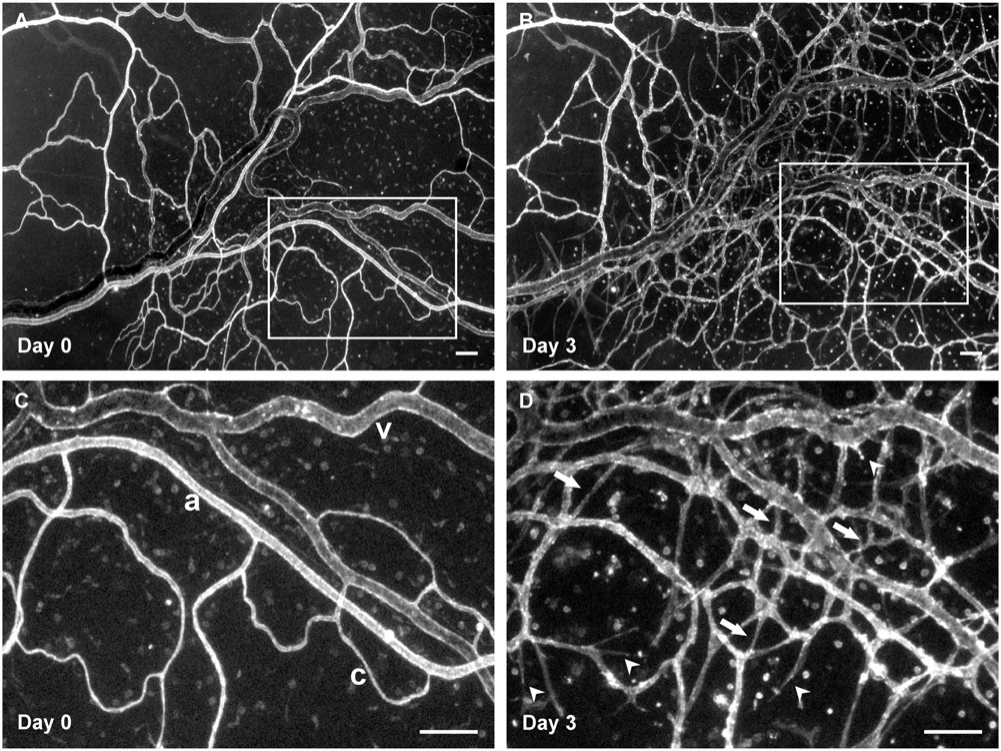
Microvascular networks in the rat mesentery culture model before and after angiogenesis. A–D) Comparison of the same network labeled with BSI-lectin on day 0 (before) and day 3 (after) post-stimulation with 10% serum identifies new vessels. C, D) Higher magnification images of the same region indicated by the above squares. Examples of arterioles, venules, and capillaries are marked by letters “a,” “v,” and “c,” respectively. Increased blood capillary sprouting is evident by day 3 (arrowheads). The new vessel segments (arrows) are indicative of increased vessel density. Lectin also labels a population of unidentified interstitial cells. Scale bars = 100μm.

**Fig 2:**
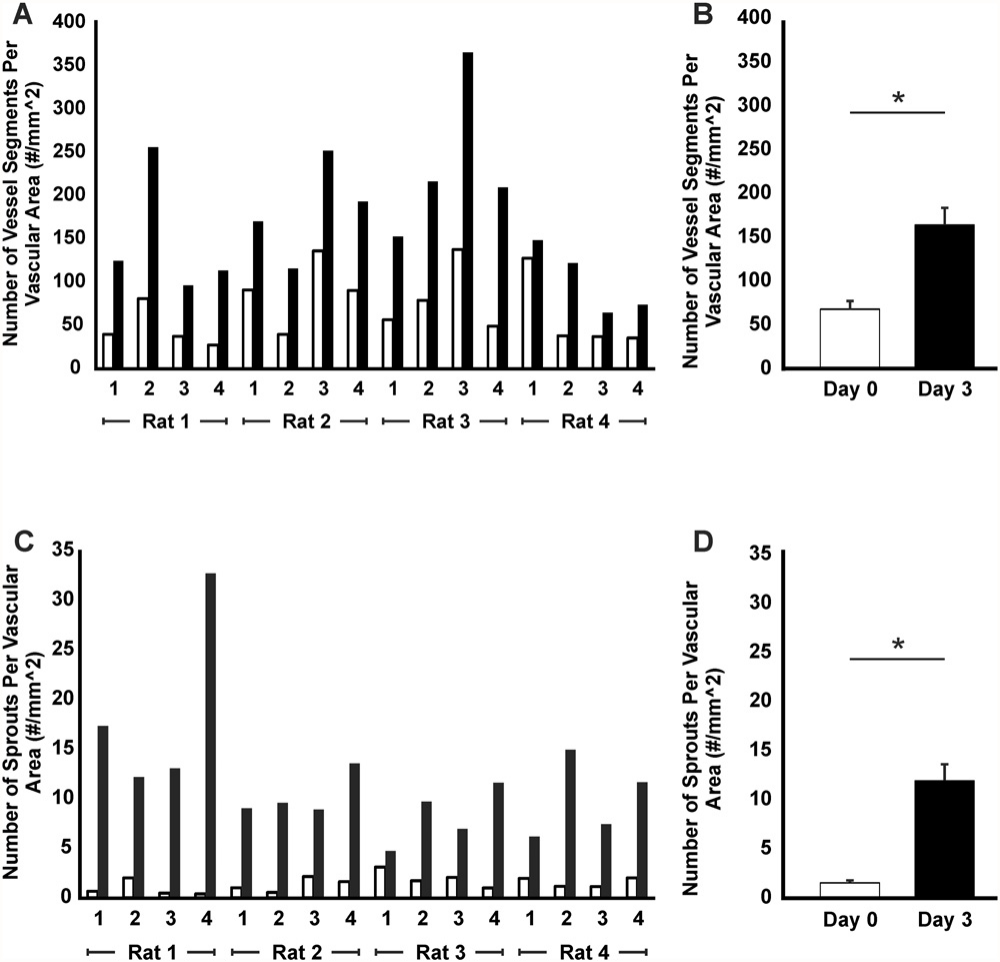
Angiogenic response in rat mesentery tissues following stimulation. Vessel density (A, B) and number of capillary sprouts (C, D) per vascular area were quantified before and after stimulation with 10% serum. An increase in both metrics occurred for each tissue. Comparison between day 0 and day 3 confirmed a significant difference in both the average number of vessel segments (p < 0.001) and the average number of sprouts (p < 0.001) per vascular area. White bars represent day 0 (before) and black bars represent day 3 (after).

**Fig 3:**
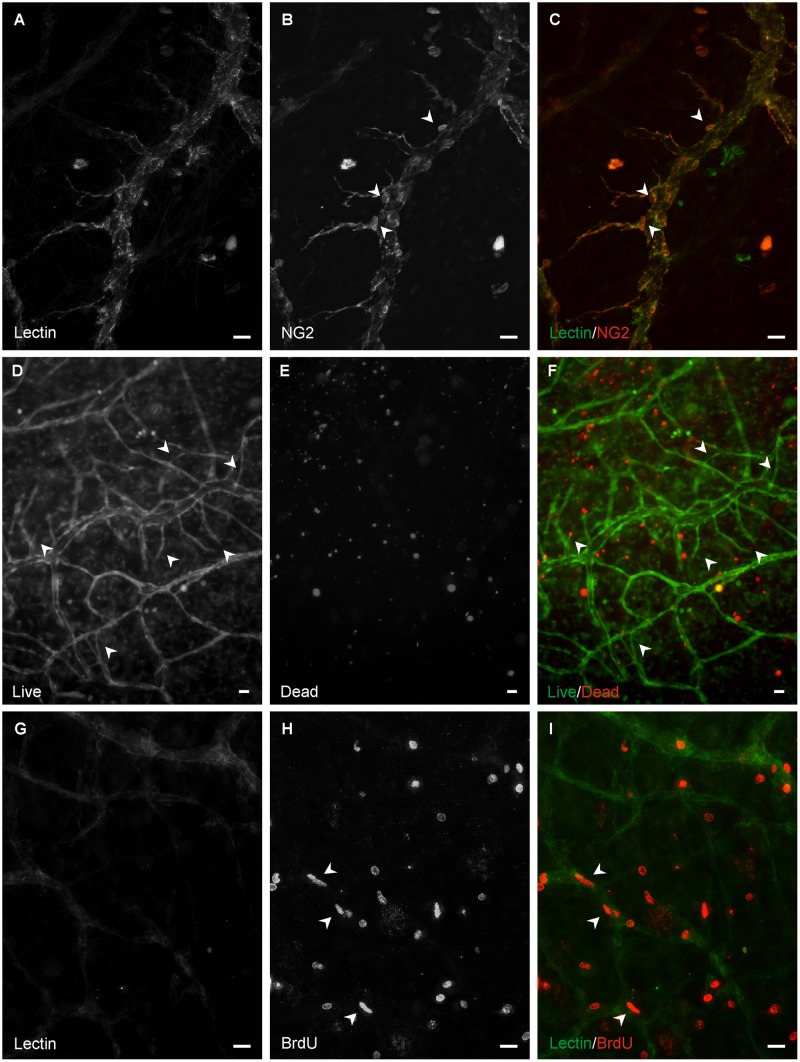
Presence of pericytes, viable cells, and proliferative cells in angiogenic microvascular networks. A–C) NG2 positive pericytes along BSI-lectin positive microvessels on day 3 post culture with 10% serum. Arrowheads show NG2+ pericytes. D–F) Live/Dead labeling of microvessels on day 3 post culture with 10% serum. Live and dead cells were identified by positive Calcein-AM and Eth-D labeling, respectively. Arrowheads indicate the Calcein-AM+ newly formed sprouts and connections within the network. G–I) BrdU positive cells along BSI-lectin positive microvessels on day 3 post culture with 10% serum. Proliferation of endothelial cells is supported by the observation of elongated nuclei within the lectin positive vessels (arrowheads). Scale bars = 20μm.

Lectin labeling at day 0 and day 3 identified endothelial cells along the hierarchy of intact rat mesenteric microvascular networks (Fig. 1). Mesenteric tissues stimulated in culture for 3 days with 10% FBS displayed dramatic increases in microvascular network growth. Qualitative comparison of network regions before and after stimulation revealed increases in vessel density and capillaries sprouting from pre-existing vessels (Fig. 1). Increases in both the number of vessel segments per vascular area and the number of capillary sprouts per vascular area were clearly evident and statistically significant (p < 0.05 for both measures) (Fig. 2). Consistent with our previous characterization of the model, angiogenic networks at day 3 contained NG2-positive pericytes along the microvessels confirming that vascular pericytes remain present along endothelial cells during angiogenesis after *ex vivo* culture (Fig. 3 A-C). In addition, vessel growth was associated with vascular cell viability (Fig. 3 D-F) and proliferation (Fig. 3 G-I).

### Time-Lapse Imaging Enables Evaluation of Anti-Angiogenic Drugs

**Fig 4:**
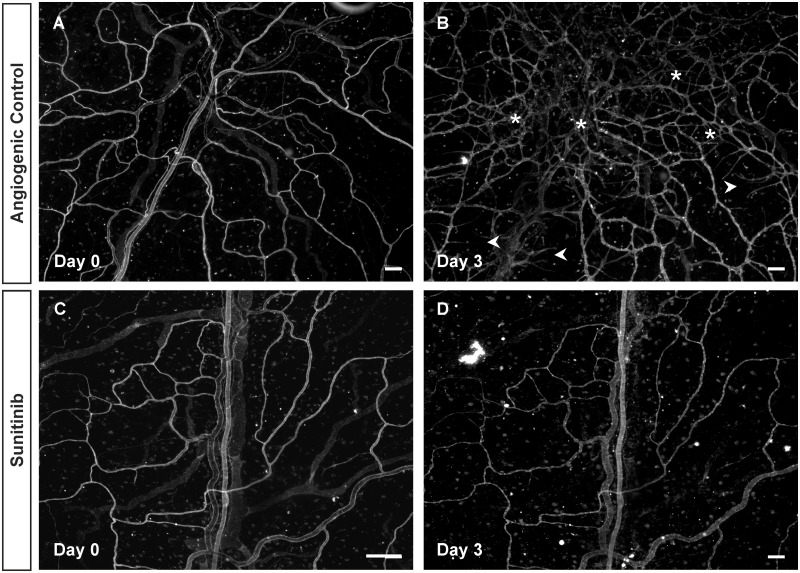
Inhibition of angiogenic response in microvascular networks by sunitinib. Examples of mesentery tissues stimulated with 10% serum for 3 days with or without sunitinib treatment. A, B) Comparison of the same network labeled with BSI-lectin on day 0 and day 3 post-stimulation with 10% serum confirms a robust angiogenic response. Asterisks represent the regions with a higher vessel density, and arrowheads point to newly formed sprouts. C, D) Comparison of another network treated with sunitinib and labeled with BSI-lectin on day 0 and day 3 post-stimulation with 10% serum. The angiogenic response observed in the control group is inhibited with sunitinib treatment. Scale bars = 100μm.

**Fig 5:**
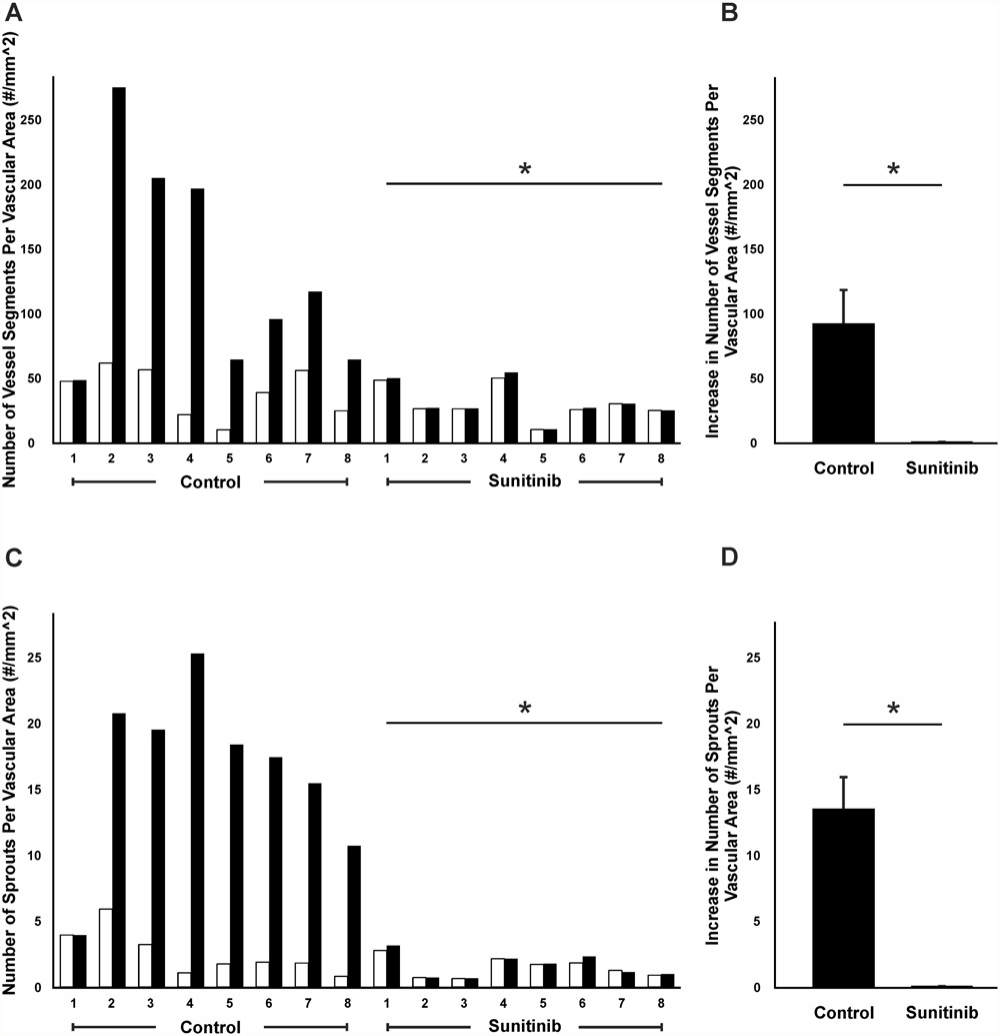
Quantification of angiogenesis inhibition following sunitinib treatment. The effect of 3-day exposure to sunitinib on 10% serum growth was evaluated based on two angiogenic metrics: Vessel density (A, B), and number of capillary sprouts (C, D) per vascular area. Control tissues were stimulated with 10% serum only. A, C) Each pair of bars represents a tissue. The average increase in vessel segments (B) and capillary sprouts (D) per area in control group is plotted against the sunitinib-treated group. * represents a significant difference between control and sunitinib groups (p < 0.05 for vessel segments and p < 0.01 for capillary sprouts). White bars represent day 0 (before) and black bars represent day 3 (after).

In order to validate the efficacy of the rat mesentery culture model for drug evaluation, we tested a known receptor tyrosine kinase inhibitor, sunitinib, and a well-known anti-VEGF drug, bevacizumab. Comparison of microvascular networks before (Day 0) and after (Day 3) enabled identification of tissue specific responses. VEGFR-2 blockage via sunitinib treatment inhibited any angiogenic response (Figs. 4 and 5). 7 out of 8 control tissues displayed a dramatic increase in vascular density and capillary sprouting post stimulation with 10% FBS. The presence of sunitinib in each of the treated tissues inhibited these responses. The mean increase in vascular density in control tissue was 92.1 vessel segments compared to 1.0 for the sunitinib treated group (p < 0.05). The mean increase in number of capillary sprouts was 13.8 vs 0.1 for control and treated tissues respectively (p < 0.01).

**Fig 6:**
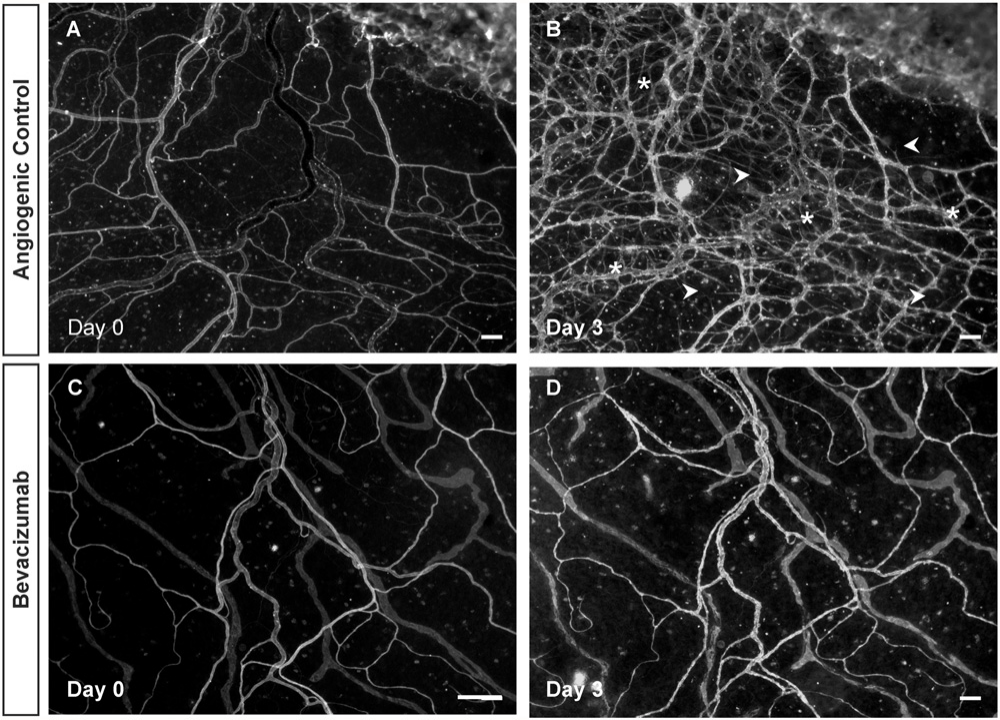
Inhibition of angiogenic response in microvascular networks by bevacizumab. Examples of mesentery tissues stimulated with 10% serum for 3 days with or without bevacizumab treatment. A, B) Comparison of the same network labeled with BSI-lectin on day 0 and day 3 post-stimulation with 10% serum confirms a robust angiogenic response. Asterisks represent the regions with a higher vessel density, and arrowheads point to newly formed sprouts. C, D) Comparison of another network treated with bevacizumab and labeled with BSI-lectin on day 0 and day 3 post-stimulation with 10% serum. The angiogenic response observed in the control group is inhibited with sunitinib treatment. Scale bars = 100μm.

**Fig 7:**
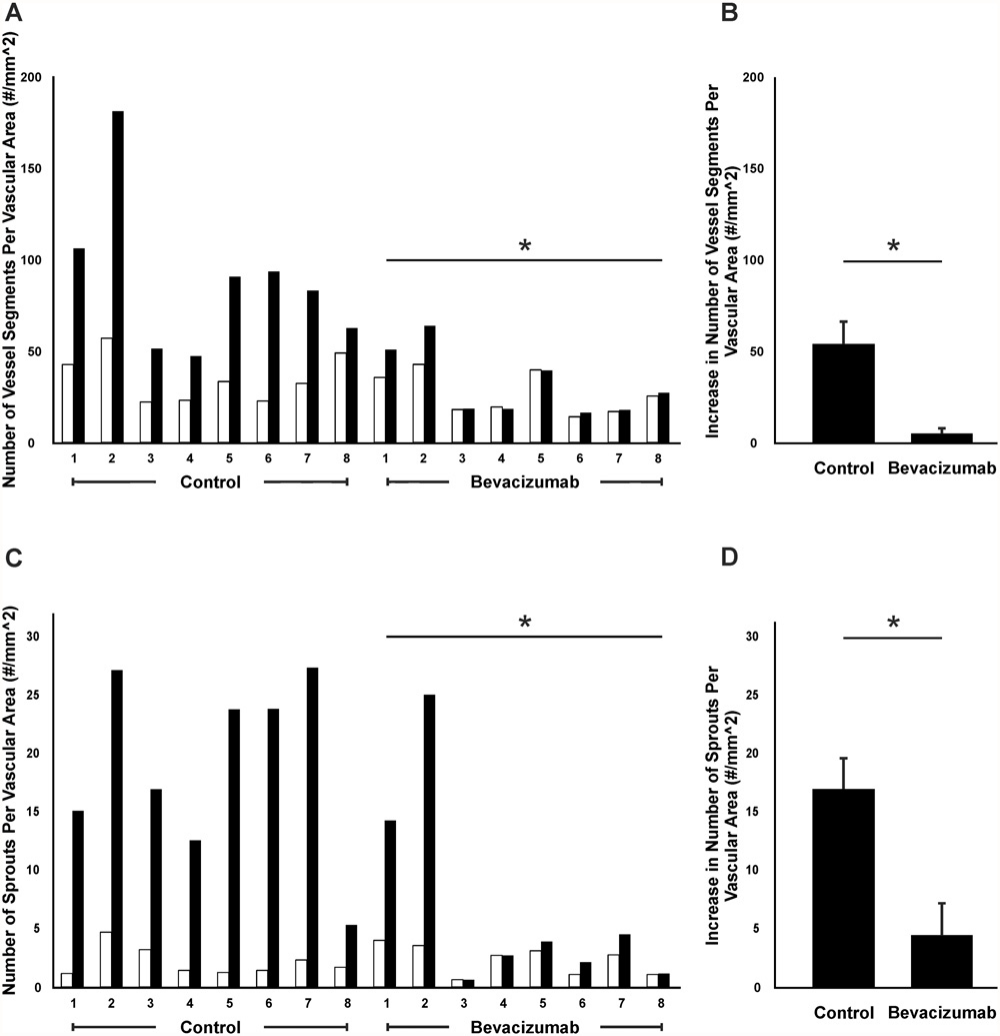
Quantification of angiogenesis inhibition following bevacizumab treatment. The effect of 3-day exposure to bevacizumab on angiogenic growth caused by 10% serum was evaluated by two angiogenic metrics: Vessel density (A, B), and number of capillary sprouts (C, D) per vascular area. Control tissues were stimulated with 10% serum only. A, C) Each pair of bars represents a tissue. The percentage of increase in vessel segments (B) and capillary sprouts (D) per area in control group is plotted against the bevacizumab-treated group. * represents a significant difference between control and bevacizumab groups (p < 0.05 for vessel segments and p < 0.05 for capillary sprouts). White bars represent day 0 (before) and black bars represent day 3 (after).

Bevacizumab treatment also served to inhibit the serum induced angiogenesis in cultured rat mesenteric microvascular networks. In this separate study, 8 out of 8 control tissues displayed a dramatic increase in vascular density and capillary sprouting post stimulation with 10% FBS (Figs. 6 and 7). Media supplementation with bevacizumab inhibited both the vascular density and sprouting responses (Fig. 7). The mean increase in vascular density in control tissue was 53.9 vessel segments compared to 5.0 for the bevacizumab treated group (p < 0.05). The mean increase in number of capillary sprouts was 16.9 vs 4.4 for control angiogenic and treated tissues respectively (p < 0.05).

## Discussion

The results of this study support the use of the rat mesentery culture model as an *ex vivo* tool for anti-angiogenic drug evaluation on intact microvascular networks based on time-lapse imaging. Our initial introduction of the rat mesentery culture model for angiogenesis research established that it could be used for probing pericyte-endothelial cell interactions [8]. Our current study expands upon our previous work and supports the imaging in the rat mesentery culture model at multiple time points as a method for quantification of tissue-specific growth responses. Our observation of pericytes and increased proliferation of endothelial cells after our imaging method supports the notion that the microvascular growth in the cultured rat mesenteric tissues involves dynamic interactions between multiple cell types.

**Fig 8:**
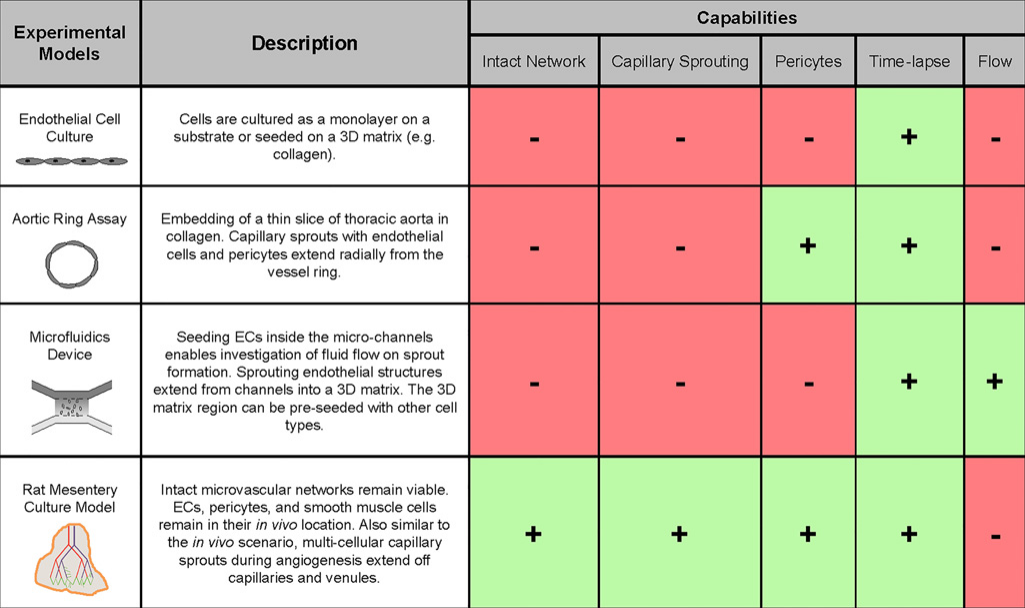
Advantages of rat mesentery culture model. Compared to other common angiogenesis assays, the rat mesentery culture model enables time-lapse imaging of capillary sprouting and investigation of pericyte-endothelial cell interaction in an intact microvascular network. Refs: Endothelial cell culture [20], Aortic ring assay [2], Microfluidic devices [3,4].

While in general rodent models and other animal models cannot substitute for human clinical trials in understanding the mechanisms involved in physiological events, they have proved to play a crucial rule in pre-clinical testing. Indeed, *in vivo* animal studies are identified as a crucial step between *in vitro* studies and clinical trials [9,18], since they offer a less expensive method for screening drug responses. And as *ex vivo* or *in vitro* models more closely mimic the complexity of *in vivo* scenarios, they too have become useful pre-clinical alternatives. Compared to commonly used tissue culture models and *in vitro* cell systems, the rat mesentery culture model is unique because growth occurs within an intact microvascular network (Fig. 8). Evaluation of responses across the hierarchy of a network is not possible with other methods. Single or co-culture cell assays are limited to observation of cell tube formation and the interconnection of multiple tubes with no hierarchical distinction. *Ex vivo* tissue explants have proven extremely useful for studying angiogenesis. Nicosia et al. first introduced the aortic ring model as an assay to investigate angiogenic sprouting from aortic segments embedded in a collagen gel [2]. While sprouting in the aortic ring involves multiple cell types, a limitation is that the sprouting occurs from macrovessels, atypical of the *in vivo* process. Also, while these sprouts are associated with perivascular wrapping cells, the vessels do not typically form networks with a hierarchical structure. More recently, the retina culture model has been introduced in which angiogenesis does occur from intact microvascular networks [19,20]. In these models, culturing tissues harvested from GFP-transgenic mice strains offers the ability for observing endothelial sprouting over time, yet compared to the mesenteric tissues, the retina is thicker and can be difficult to prepare. We hypothesize that the maintained three-dimensional complexity in the rat mesentery culture model offers an advantage over less complicated systems. For example, work by others highlights the value of more realistic three-dimensional microfluidic environments that integrate tumor spheroids and endothelial monolayers. Compared to less complicated two-dimensional assays, the more realistic environment influenced the effective dose required for inhibiting cancer cell dispersion [21]. Yet in microfluidic models, single channels are typically lined with just endothelial cells and the pattern of an intact network is lost. Thus, the use of the rat mesentery model offers a new platform for assays, since the physiological network pattern of arterioles, venules, and capillaries is maintained in rat mesentery model. Our results in the current study motivate future studies to compare responses between the *in vivo* scenario, the rat mesentery culture model, and the other current models from the literature with the question remaining—does the maintained complexity of the rat mesentery culture model more closely characterize the *in vivo* scenario? Future studies will also be required to determine how long the *in vivo* like intact network complexity is maintained in culture. Based on previous characterization of the rat mesentery culture model, we know that tissues remain viable beyond seven days [8].

The rat mesentery culture model is advantageous because it can be used for 1) real-time imaging within the same tissue, and 2) quantification of endothelial cell sprouting at specific locations within a microvascular network during growth factor-induced angiogenesis. Another key advantage of our model is its simplicity—the tissue is easy to obtain, self-contained and does not need to be embedded. The current study expands the capabilities of the model and introduces it as a novel drug screening platform. The value of the rat mesentery for chemotherapy and anti-angiogenic drug testing is validated by numerous chronic *in vivo* animal studies [22,23]. Our study serves to expand the usefulness of this tissue by demonstrating the ability to evaluate drugs in an *ex vivo* scenario, which is more applicable for high content pre-clinical screening. Both bevacizumab and sunitinib are established anti-angiogenic drugs that target VEGF or its receptors on endothelial cells. Our results show that these agents are successful in overall inhibiting angiogenesis within our cultured networks. The variability in the responses to both drug treatments highlights the potential tissue specific responses, which were discussed above in the context of the angiogenesis responses to serum. Response heterogeneity can also be appreciated within a specific network (Fig. 1). Angiogenesis is local regions of a network is consistent with *in vivo* observations made in the same tissue following inflammatory stimulation of angiogenesis [8,11,24] and, while such variability could be viewed as problematic, we speculate that future studies utilizing the rat mesentery culture model might be able to provide valuable information about the local cues and their causality in creating differences across a network. In addition, from a drug testing perspective, we speculate it would be useful to know where angiogenesis is stimulated within a network. Future studies will be needed to compare how the variability and dose sensitivity for the rat mesentery culture model compares to other, less complex angiogenesis assays. Similar to other *ex vivo* culture systems, a limitation of the rat mesentery culture model is that it lacks the presence of blood flow and related effects of shear stress, which had been shown to influence angiogenesis *in vivo* [25,26]. The recent development of microfluidic-based assays has emphasized the importance of including shear stress in *in vitro* models [3,4,27,28]. While future developments of the rat mesentery culture model will be needed to include flow, we believe that the top-down approach to maintain the multi-cell and multi-system complexity of an intact microvascular network might enable unique applications exemplified by our drug testing results.

Our drug treatment results support the use of the rat mesentery culture model for drug testing. For these studies, serum-stimulated tissues were used as our control group because we were interested in showing the anti-angiogenic effect of drugs, and based on results shown in Fig. 2, 10% FBS stimulation caused robust microvascular growth compared to tissues cultured for 3 days in serum-free media. As an indicator of model robustness, 31 out of 32 serum-stimulated tissues from the three separate studies displayed a dramatic response. Moreover, the comparison of images before and after treatment reduces issues of variability that might influence non-paired statistical analysis. The explant specific responses to serum-induced angiogenesis varied from 16% to 320% increases in vessel density and from 50% to 8000% increases in sprout density. Although the low number of existing sprouts in tissues before stimulation could be an explanation for some of the more drastic increases in the number of sprouts, the specific causes for this variation remain unknown. Nevertheless, the ability to quantify growth in the same tissue provides definitive evidence of whether a response occurred and enables the application of appropriate analytical methods to account for random variation among samples. Tissue specific responses were also present in our angiogenesis control groups for the sunitinib and bevacizumab studies. While an explanation of occasional lack of responses is unclear, the concept of non-responders is consistent with other *ex vivo* models [29,30], and we speculate it might be associated with starting network size.

Unfortunately, the mouse mesentery is avascular [9], eliminating the substitution of GFP-transgenic mice tissue for rat mesentery, as utilized so far in our model. While future studies are necessary to determine the possibility of observing angiogenic growth into the avascular murine mesenteric tissues, we show that a simple lectin labeling of the rat mesentery cultures is sufficient to determine network growth at different time points. We selected FITC conjugated lectin for this study because it identifies vessels along the hierarchy of rat mesentery networks, including capillary sprouts. The use of fluorochromes alternative to FITC could 1) prevent the need to re-label at later time points, and 2) enable the identity of the day 0 (starting) cell population versus the day 3 new cell population attributed to vessel growth. An alternative approach, that also warrants future investigation, would be cell-targeted transfections. Such an approach would also enable the higher time-resolution tracking of specific cell types across a network related to the incorporation and growth of the new vessels. Interestingly, previous trial experiments with the Alexa fluorochromes (data not shown) in the laboratory indicate that re-labeling after 3 days is still necessary. These observations suggest that in addition to fluorochrome labeling, lectin binding might also be transient. Nonetheless, the comparison between the before and after images in our study remains to be sufficient for identifying new vessel segments and the evidence of proliferation in our model suggests that these new segments are formed by new cells.

The simplicity of the model is supported not necessarily by the number of steps, yet rather by the lack of tissue manipulation required to maintain the network organization complexity. Consequently, we feel the simplicity of our model is appreciated by considering the fact that each step is relatively not time-intensive and that the model allows an unmatched multi-cellular network view. Since each tissue is self-contained, one essentially has to harvest it from the animal and place it into a culture dish. No matrix embedding, cell passaging, cell isolation, cell seeding, additional tissue handling or fabrication steps are required.

In summary, this study, for the first time, introduces a novel *ex vivo* method to evaluate an anti-angiogenic drug based on time-lapse imaging on an intact microvascular network. A challenge in evaluating angiogenesis therapies is the lack of realistic *in vitro* models. Our results meet this challenge and offer a simple and reproducible *ex vivo* assay to measure the effects of possible anti-angiogenic agents on vessel density and capillary sprouting across the hierarchy of networks. We also envision the use of tissues harvested from pathological rat strains will enable the investigation of how anti-angiogenic therapies are influenced by disease states.
